# KSHV ORF20 Promotes Coordinated Lytic Reactivation for Increased Infectious Particle Production

**DOI:** 10.3390/v16091418

**Published:** 2024-09-05

**Authors:** Odelia Orbaum-Harel, Anna Sloutskin, Inna Kalt, Ronit Sarid

**Affiliations:** 1 The Mina and Everard Goodman Faculty of Life Sciences, Bar-Ilan University, Ramat Gan 5290002, Israel; del.orbaum@gmail.com (O.O.-H.);; 2 Advanced Materials and Nanotechnology Institute, Bar-Ilan University, Ramat Gan 5290002, Israel

**Keywords:** Kaposi’s sarcoma-associated herpesvirus, KSHV, open reading frame 20, ORF20, lytic reactivation, unique long 24, UL24

## Abstract

Kaposi’s sarcoma-associated herpesvirus (KSHV) is a cancer-causing virus that establishes life-long infection. KSHV is implicated in the etiology of Kaposi’s sarcoma, and a number of rare hematopoietic malignancies. The present study focuses on the KSHV open reading frame 20 (ORF20), a member of the conserved herpesvirus UL24 protein family containing five conserved homology domains and a conserved PD-(D/E)XK putative endonuclease motif, whose nuclease function has not been established to date. ORF20 encodes three co-linear protein isoforms, full length, intermediate, and short, though their differential functions are unknown. In an effort to determine the role of ORF20 during KSHV infection, we generated a recombinant ORF20-Null KSHV genome, which fails to express all three ORF20 isoforms. This genome was reconstituted in iSLK cells to establish a latent infection, which resulted in an accelerated transcription of viral mRNAs, an earlier accumulation of viral lytic proteins, an increase in the quantity of viral DNA copies, and a significant decrease in viral yield upon lytic reactivation. This was accompanied by early cell death of cells infected with the ORF20-Null virus. Functional complementation of the ORF20-Null mutant with the short ORF20 isoform rescued KSHV production, whereas its endonuclease mutant form failed to enhance lytic reactivation. Complementation with the short isoform further revealed a decrease in cell death as compared with ORF20-Null virus. Finally, expression of IL6 and CXCL8, previously shown to be affected by the hCMV UL24 homolog, was relatively low upon reactivation of cells infected with the ORF20-Null virus. These findings suggest that ORF20 protein, with its putative endonuclease motif, promotes coordinated lytic reactivation for increased infectious particle production.

## 1. Introduction

Kaposi’s sarcoma (KS) was first described in 1872 by Moritz Kaposi [1]. The virus involved in KS, known as Kaposi’s sarcoma-associated herpesvirus (KSHV), or human herpesvirus 8 (HHV-8), belongs to the Gammaherpesvirinae subfamily and is one of only a few oncogenic human viruses [2]. KSHV is also etiologically linked with two other hematopoietic disorders: primary effusion lymphoma (PEL) and multicentric Castleman’s disease (MCD). In addition, KSHV is associated with a systemic inflammatory condition, termed KSHV-associated inflammatory cytokine syndrome (KICS), which clinically resembles MCD but lacks pathologic nodal changes [3,4,5,6,7,8,9,10,11]. The KSHV genome comprises a double-stranded DNA of approximately 165-kbp, encoding more than 85 open reading frames (ORFs) and a set of non-coding RNAs, including micro, long, and circular RNAs [3,12,13]. Like other herpesviruses, KSHV exhibits a two-phase infection cycle: a lytic phase, characterized by an extensive expression of viral genes along with replication of the virus genome, and a latent phase, which involves the presence of chromatin-coated circular viral genomes in the cell nucleus, with the ongoing expression of a limited set of viral genes, and no virus propagation. Accordingly, KSHV genes can be classified into two categories: lytic and latent genes. The lytic genes are further classified into three groups: immediate-early (IE) genes, which primarily promote viral infection mostly through controlling the expression of selected host and early viral genes; early (E) genes, which encode proteins involved in metabolism, viral genome replication, and control of intracellular processes, such as DNA replication and protein translation; and late (L) genes, which are expressed after replication of the viral genome, and include structural viral proteins [14,15,16]. Nevertheless, most KSHV infections are asynchronous, and the conventional categorization of viral gene expression is not always justified. In addition, the expression kinetics of certain genes may vary depending on the infected cell type [17,18].

The KSHV ORF20 is a member of the UL24 protein family, which is conserved in all three subfamilies of Herpesviridae [19]. The universal presence of UL24 suggests a fundamental role of this protein in virus infection, yet our current understanding of the function of KSHV ORF20 is limited. Of note, ribosomal footprinting data and in silico prediction revealed the presence of initiating ribosomes at three distinct ORF20 positions, enabling the synthesis of three colinear protein isoforms of ORF20. However, the potential mechanisms governing their differential expression and their respective functions are yet to be clarified [20]. KSHV ORF20 induces G2/M cell cycle arrest, followed by apoptosis in both human and mouse cells, and this is correlated with a concomitant inactivation of the kinase activity of the mitotic complex Cyclin B/Cdc2 [21]. A similar activity has also been demonstrated for the murine gammaherpesvirus 68 (MHV68) ORF20 gene product, as well as for other UL24 homologs, including UL24 encoded by herpes simplex virus type 1 (HSV-1) and the UL76 gene product encoded by human cytomegalovirus (hCMV) [21,22,23]. A number of potential interacting partners of ORF20 have been identified through affinity purification of ectopically expressed ORF20, coupled to mass spectrometry. This includes a large number of 40S and 60S ribosomal subunit proteins and the interferon-stimulated gene (ISG) product oligoadenylate synthetase-like protein (OASL), which was shown to enhance KSHV infection in an ORF20-dependent manner [24,25]. Both ORF20 and OASL co-purified with 40S and 60S ribosomal subunits and were associated with polysomes when co-expressed; however, neither ORF20 nor OASL exhibit a global effect on translation, suggesting that these proteins may control the expression of a subset of proteins [25]. A more recent study utilized a promiscuous biotin ligase proximity labeling method to identify the proximal interactome of ORF20 during lytic reactivation, revealing a number of protein partners, including ribosomal proteins and the KSHV DNA processivity factor, ORF59 [26].

Studies on UL24 homologs can provide insights into the potential function of KSHV ORF20. For instance, HSV-1 UL24 has been reported to disperse Nucleolin and B23 from the nucleolus during HSV-1 infection [27,28]. It has also been shown to negatively affect viral gene expression [29], and to inhibit cGAS-STING-mediated activation of IFN [30]. Similarly, expression of UL76 inhibits hCMV production, and represses its replication and virus propagation [31]. Additionally, UL24 affects the subcellular distribution of viral envelope glycoproteins that are involved in fusion at late stages of infection, and this involvement suggests a role for UL24 in virion morphogenesis or in viral egress [32]. UL24 was also shown to inhibit the activation of nuclear factor kappa B (NF-κB) in the DNA sensing signaling pathway via binding to the Rel homology domains (RHDs) of the NF-κB subunits p65 and p50 and abolishing their nuclear translocation [30]. Further functions have been identified for UL76, including inducing host chromosomal aberrations [23], regulating UL77 gene expression [33], reducing pre-rRNA transcription [34], and eliciting aggresome formation via interaction with S5a of the ubiquitin proteasome system [35]. Pseudorabies (PRV) UL24 antagonizes the cGAS-STING signal pathway through IRF7 degradation [36], impairs RIG-I signaling [37], inhibits the TNF-α-mediated NF-κB signaling pathway through p65 degradation [38], and suppresses the transcription of ISG20, thereby antagonizing its antiviral effect [39]. Further domain mapping analysis showed that the N-terminus (amino acids 1–90) of PRV UL24 is responsible for the inhibition of ISG20 transcription [39]. Additional analysis demonstrated that the antiviral activity of porcine ZCCHC3, which hinders PRV proliferation by modulating innate immune responses at the cellular level, is counteracted by PRV-encoded UL13 and UL24 proteins, thereby undermining its ability to combat the virus [40].

In the present study, in order to better understand the functions of KSHV ORF20, we generated a recombinant KSHV genome that fails to express all ORF20 isoforms and characterized its infection cycle. Our findings reveal that lytic reactivation of ORF20-Null viruses results in an accelerated accumulation of viral lytic transcripts and proteins along with early cell death compared to wild-type (WT) viruses. This is coupled with an increase in viral DNA copies and a reduction in infectious viral particle production. Notably, we show that the short isoform of ORF20, containing a conserved putative endonuclease motif, is significant for efficient lytic virus reactivation and virion production.

## 2. Materials and Methods

### 2.1. Reverse-Transcription (RT)-Quantitative PCR (RT-qPCR)

RNA extraction, cDNA synthesis, and reverse transcription-quantitative PCR (RT-qPCR) were performed as previously described [41]. The expression levels obtained for each gene were normalized to those of GAPDH. The fold change in expression was calculated by comparing the level of expression to the normalized value for the wild-type virus at 24 h post induction. The primers used were the following: ORF19, 5′-ATACCAGGTTCAAGCGGCG-3′ and 5′-TGGATTGCTGGAGTTTGGG-3′; ORF20, 5′-CCGATCTATGGCGG TTTCTAAG-3′ and 5′-CCCACCCGATACCAGAATTAC-3′; ORF21, 5′-CGTAGCCGACGCGGATAA-3′ and 5′-TGCCTGTAGATTTCGGTCCAC-3′; ORF45, 5′-CTAGCACA CACGATGAAGAGAG-3′ and 5′-GGAAGTGATGAAAGAGGTGGAG-3′; ORF59, 5′-CGAGTCTTCGCAAAAGGTTC-3′ and 5′-AAGGGACCAACTGGTGTGAG-3′; ORF65, 5′-ATATGTCGCAGGCCGAATAC-3′ and 5′-CCACCCATCCTCCTCAGATA-3′; IL6, 5′-GGATTCAATGAGGAGACTTGCC-3′ and 5′-ACAGCTCTGGCTTGTTCCTCAC-3′; CXCL8, 5′-GAACTGAGAGTGATTGAGAGTGG-3′ and 5′-CAGAGCTCTCTTCCA TCAGAAAG-3′; and GAPDH, 5′-GAAGGTGAAGGTCGGAGTC-3′ and 5′-GAAGATG GTGATGGGATTTC-3′. All PCRs were run in triplicate on a StepOnePlus real-time PCR system (Applied Biosystems Inc., Carlsbad, CA, USA).

### 2.2. Construction of ORF20-Null Recombinant KSHV Genome and Virus Reconstitution

Recombinant full-length KSHV bacterial artificial chromosome (BAC) 16 which constitutively expresses GFP under the control of the cellular elongation factor 1-alpha (EF-1α) was previously described (kindly provided by Prof. Jae Jung) [42]. BAC16, in which mCherry replaces GFP fluorescent protein, was previously generated in our lab [43]. Construction of the recombinant virus BAC16-ORF20-Null was performed using *Escherichia coli* GS1783 bacteria with a two-step recombination protocol, as described previously [44]. In contrast to our previously reported recombinant genomes [41,45,46], the construction of ORF20-Null genome comprised two rounds of two-step recombinations: the first led to the deletion of 200 bp, while the second resulted in the insertion of a synthetic cassette containing the same deleted 200 bp with selected mutations as follows: a termination codon replacing both Tyr2 and Glu69 (TAC to TAG, GAG to TAG), and a mutation replacing Leu24 with Glu (CTG to GAA). Accordingly, PCR was first used to generate an insertion cassette encoding a kanamycin resistance gene flanked with an I-SceI endonuclease recognition site and homologous ends using the pEGFP-N1 plasmid (Clontech) as a template. The primer set used to amplify this fragment was: Forward: 5′TCTTTTGCGTGCTGCTGGAAGCCTGCTCAGGGATTTCTTAACCTCGGCCTtagggataacagggta atAGGTGGCACTTTTCGGGGAAA-3′ (the first 50-nt complemented the sequences upstream to ORF20; I-Sce-I restriction site is in lower case; the kanamycin resistance gene sequence is underlined) and 5′-ATTAAATGCCACGGCTTGCTGGTCGGGACGGTGGGCACGTCTATATGTAaggccgaggttaagaaatccctgagcaggcttccagcagcacgcaaaagaT TTATTGCCGTCATAGCGCG-3′ (the first 50-nt complemented the sequences within ORF20; the next 50-nt complemented the Forward primer and is shown in lower case; the kanamycin resistance gene sequence is underlined). Integration of the resulting PCR cassette was verified by PCR and restriction enzyme digestion. The integrated cassette was then cleaved upon treatment with 1% L-arabinose, enabling a second recombination event between duplicated sequences yielding kanamycin sensitive colonies containing 200 bp deletion within ORF20. Subsequently, we generated an identical synthetic insertion cassette containing the mutations indicated above (Invitrogen, Waltham, MA, USA), and a second two-step red-recombination event was performed and validated by restriction enzyme digestion. The inserted fragment was sequenced to confirm the presence of the desired mutations. BAC DNA was purified using a large construct kit (Qiagen, Valencia, CA, USA).

### 2.3. BAC16-ORF20-Null Virus Reconstitution and Lytic Reactivation

BAC16 and BAC16-ORF20-Null reconstitution was performed as previously described [41,47]. Briefly, BAC DNA was transfected into HEK-293T cells using the Lipofectamine 2000 transfection reagent (Invitrogen, Carlsbad, CA, USA), and transfected cells were selected with 200 μg/mL hygromycin B (A. G. Scientific Inc., San Diego, CA, USA). Following selection, these cells were subcultured and mixed with iSLK cells. Lytic reactivation was induced using recombinant baculovirus that constitutively expresses RTA (BacK50) (kindly provided by Prof. David Lukac) [48] and 1 mM sodium butyrate (Sigma, St. Louis, MO, USA). BAC16-infected iSLK cells were selected with medium containing 250 μg/mL G418, 1 μg/mL puromycin, and 600 μg/mL hygromycin B. To induce lytic reactivation, infected iSLK cells were treated with 1 μg/mL doxycycline and 1 mM sodium butyrate. Virion collection involved centrifugation to remove cell debris (700× *g* for 5 min at 4 °C) followed by filtration through 0.45 μm filters (Corning, Oneonta, NY, USA) and centrifugation (40,000× *g* for 2 h at 4 °C).

### 2.4. Cell Culture

Human epithelial kidney HEK-293T cells, renal cell carcinoma SLK, and iSLK cells (kindly provided by Don Ganem, Howard Hughes Medical Institute, UCSF, San Francisco, CA, USA, and Rolf Renne, University of Florida, Gainesville, FL, USA) [49,50] were grown in Dulbecco’s modified Eagle’s medium (DMEM) (Biological Industries, Beit HaEmek, Israel) containing 50 IU/mL penicillin and 50 μg/mL streptomycin (Biological Industries, Beit HaEmek, Israel), and supplemented with 10% heat-inactivated fetal calf serum (FCS) (Biological Industries, Beit HaEmek, Israel). iSLK cells were grown in the presence of 250 μg/mL G418 and 1 μg/mL Puromycin (A.G. Scientific Inc., San Diego, CA, USA) to maintain the Tet-on transactivator and the RTA expression cassette, respectively. The growth medium of BAC16-infected HEK-293T and iSLK cells was supplemented with 200 and 600 μg/mL hygromycin (MegaPharm, San Diego, CA, USA), respectively, to maintain KSHV episomes. Viral DNA replication was inhibited using 0.5 mM phosphonoacetic acid (PAA) (Sigma). Transient transfections of plasmid DNA employed the calcium phosphate precipitation method.

### 2.5. Western Blot Analysis and Antibodies

Cells were washed twice in cold phosphate-buffered saline (PBS), suspended in radio-immunoprecipitation assay (RIPA) lysis buffer containing PMSF and a protease inhibitor cocktail (cOmplete, Roche Diagnostics, Mannheim, Germany), and incubated on ice for 25 min. Cell debris were removed by centrifugation at 13,300× *g* for 15 min at 4 °C. Sodium dodecyl sulfate (SDS) loading buffer was added, and the samples were boiled for 5 min. Protein lysates were resolved by SDS-PAGE and transferred to nitrocellulose membranes using Trans-blot Turbo RTA Midi Nitrocellulose Transfer Kit (Bio-Rad, Berkeley, CA, USA). The protein content of the different samples was verified by Ponceau S staining. Nitrocellulose membranes were blocked with 5% dry milk in Tris-buffered saline (TBS), and subsequently incubated with primary mouse antibodies to Tubulin (catalog no. E7-S; DSHB), HA (catalog no. 901515; BioLegend, San Diego, CA, USA), ORF45 [51], ORF K8 [52] (kindly provided by Prof. Yan Yuan), RTA [53] (kindly provided by Keiji Ueda), or ORF65 [54] (kindly provided by Shou Jiang Gao). Immunoreactive bands were detected using anti-mouse or anti-rabbit antibodies conjugated to horseradish peroxidase (Jackson ImmunoResearch Laboratories, Inc., West Grove, PA, USA) and visualized using an EZ enhanced chemiluminescence (ECL) detection kit (Clarity Western ECL Substrate, BioRad, Hercules, CA, USA).

### 2.6. Purification and Quantification of Viral DNA by TaqMan Real-Time PCR

Total DNA was extracted by EZ-DNA Total DNA Isolation kit (Biological Industries, Beit HaEmek, Israel). KSHV DNA was quantified by using a TaqMan-based real-time PCR assay with FAM-labeled fluorescent ORFK6 5′-CGCCTAATAGCTGCTGCTACGG-3′; Reverse: 5′-TGCATCAGCTGCCTAACCCAG-3′; Probe: 5′-CACCCACCGCCCGTCCAAATTC-3′) primers along with the cellular Cy5-labeled ERV3 gene primers (Forward: 5′-CATGGGAAGCAAGGGAACTAATG-3′; Reverse: 5′-CCCAGCGAGCAATACAGAA TTT-3′; Probe: 5′-TCTTCCCTCGAACCTGCACCATCAAT-3′) (Sigma). The total volume of the PCR reaction was 20 μL, and the reaction included 40 amplification cycles of 95 °C for 15 s and 60 °C for 45 s. Efficiency of the reaction was determined by the slope of the calibration curve. PCR reactions were run in triplicates on CFX96 Touch Real-Time PCR Detection system (Bio-Rad, Berkeley, CA, USA).

### 2.7. Infectious Virus Quantification

To quantify the infectious KSHV virions, 1.4 × 10^5^ SLK cells were seeded in 12-well plates and infected with different volumes of concentrated virions after 24 h, employing 1 h spinoculation at 1500× *g* at 25 °C in the presence of 8 µg/mL Polybrene, followed by 1 h incubation at 37 °C. After incubation, medium was replaced with DMEM and 5% FCS. Cells were then trypsinized (Biological Industries, Beit HaEmek, Israel) at 48 h post infection, washed with PBS, and fixed in 2% formaldehyde in PBS for 20 min at 4 °C. Cells were washed with PBS, and mCherry-positive cells were quantified by fluorescence-activated cell sorting (FACS) (BD LSRFortessa™, BD Biosciences, Milpitas, CA, USA). Assuming one infectious particle generates a single mCherry-positive cell, the titer of infectious units (IU) was calculated as a percentage of the number of uninfected cells, according to Poisson’s law and expressed as IU per milliliter.

### 2.8. Cell Death Analysis

For cell death assay, iSLK-WT and iSLK-ORF20-Null cells were seeded in 24-well plates. After 24 h, cells were treated to induce lytic reactivation and further incubated for 24, 48, 72, and 96 h. Supernatant was collected, cells were trypsinized and collected. Cell viability was determined with trypan blue dye (Sigma, Darmstadt, Germany) by the Countess II Automated Cell Counter (Thermo Fisher, Waltham, MA, USA).

### 2.9. Recombinant Lentiviral Vectors

The lentiviral vector DsRed (kindly obtained from Dr. Noam Stern-Ginossar), which enables expression of a gene of interest under the control of the IE-CMV promoter along with downstream IRES-dependent ZsGreen fluorescent protein, was utilized for the expression of C-terminal HA-tagged ORF20 isoforms. DNA inserts were synthesized through PCR amplification using BAC16 DNA as template along with the following primers: Full length (FL)—Forward: AAAGCGGCCGCGCCACCATGTACGAGGTTTTTACAGACTTTC Reverse: AAAGGATCCTTAAGCGTAATCTGGAACATCGTATGGGTATGGACCTGAACAAGCCGGTCCAGC; Short (S)—Forward: AAAGCGGCCGCGCCATGGTACGTCCAACCGAGGCCGAGG Reverse: AAAGGATCCTTAAGCGTAATCTGGAACATCGTATGGGTATGGACCTGAACAGCCGGTCCAGC. The Short endonuclease mutant (ORF20-SEM E154A/K156A) was generated using two rounds of amplification, the first involving the use of flanking outer primers, described for S, with overlapping inner sense and anti-sense primer pairs. This was followed by a second round of amplification using the initial PCR products and the outer primers. Forward (inner primer): CGTTGTAGCACTCGCAACTTGTCTG Reverse (inner primer): CAGACAAGTTGCGAGTGCTACAACG. All constructs were verified by sequencing.

Recombinant lentiviral vectors were generated through the transfection of lentiviral vectors along with Gag-Pol-Rev and VSV-G-Env expression plasmids into HEK-293T cells via the calcium phosphate precipitation method. Supernatants were collected 48–72 h after the transfection, and viral titers were determined as described for KSHV using ZsGreen fluorescent protein as a marker.

## 3. Results

### 3.1. Alignment of KSHV ORF20 with UL24 Family Members Encoded by Human Herpesviruses

**Figure 1:**
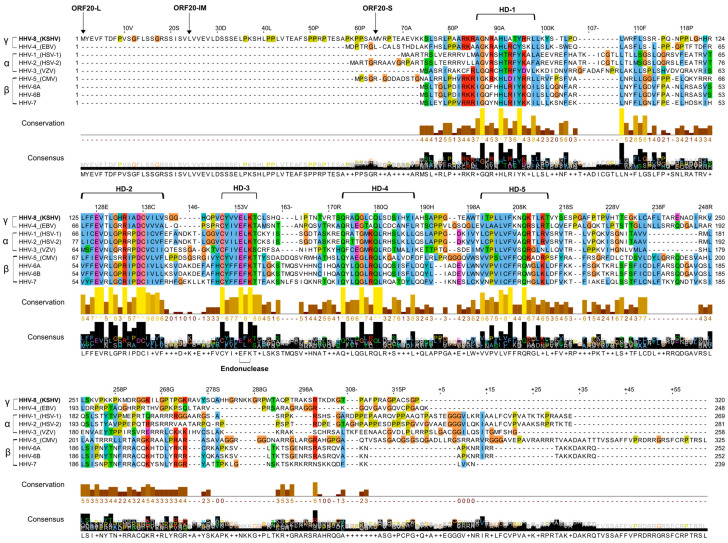
Multiple amino acid sequence alignment of UL24 family proteins encoded by human herpesviruses. The sequence alignment displays UL24 family proteins encoded by human herpesviruses 1–8 belonging to the α, β, and γ subfamilies of the Herpesviridae family (HSV-1 UL24 [GenBank: AJE60397.1], HSV-2 UL24 [NCBI reference sequence: YP_009137175.1], VZV ORF35 [NCBI reference sequence: NP_040158.1], EBV BXRF1 [GenBank: QAO57367.1], hCMV UL76 [GenBank: AAS48967], HHV-6A U49 [GenBank: AKZ18156.1], HHV-6B U49 [NCBI reference sequence: NP_050230.1], HHV-7 UL24 [NCBI reference sequence: YP_073789.1], and KSHV ORF20 [GenBank: AAB62654.1]. Analysis employed ClustalO [57] for multiple sequence alignment, and Jalview [58] for output editing. The initiation positions of the three KSHV ORF20 isoforms, ORF20-FL, ORF20-IM, and ORF20-S, are indicated by arrows, while conserved homology domains (HD) 1–5, and the conserved endonuclease motif are also marked.

KSHV ORF20 is a member of the conserved herpesvirus UL24 protein family, which is encoded by all herpesviral subfamilies. This family includes UL24 encoded by HSV-1/2 and PRV, ORF35 encoded by varicella-zoster virus (VZV), UL76 encoded by hCMV, U49 encoded by human herpesvirus 6 and 7 (HHV-6/HHV-7), BXRF1 encoded by Epstein–Barr virus (EBV), and ORF20 encoded by MHV68. Transcriptional profiling of KSHV-infected iSLK cells in which lytic viral replication was induced, along with in silico prediction, previously revealed three colinear isoforms of KSHV ORF20 protein of 320-, 297-, and 257-amino acids (aa) [20,25]. Two isoforms, termed full length (ORF20-FL) and short (ORF20-S), initiate translation with a Methionine start codon, whereas translation of the intermediate isoform (ORF20-IM) utilizes a CTG non-canonical start codon, encoding Leucine. The UL24 protein family shares five homology domains (HDs) [55], and all, including KSHV ORF20, contain a predicted conserved PD-(D/E)XK endonuclease motif found in a large superfamily of restriction endonuclease-like proteins (Figure 1) [19]. The function of this motif is currently unknown, and endonuclease activity has not yet been proven in any of the UL24 family proteins. Nonetheless, mutation of the endonuclease motif largely reduces HSV-1 replication and thus hints at its requirement for productive infection [56]. Of note, ClustalO alignment [57] of the UL24 protein family indicated that the homology is mostly of ORF20-S while the 63 and 23 N-terminal amino acids, included in the FL and IM isoforms, respectively, are unique to KSHV ORF20.

### 3.2. Kinetics of ORF20 Gene Expression

**Figure 2:**
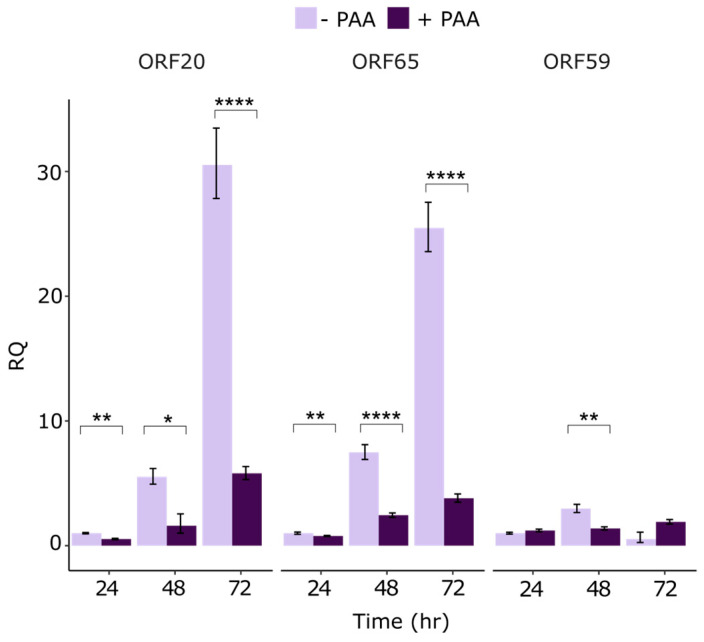
Kinetics of KSHV ORF20 gene expression following lytic reactivation. WT BAC16-infected iSLK cells were treated with doxycycline and n-Butyrate in the absence or presence of 0.5 mM PAA. Cells were collected at 24, 48, and 72 h post induction. RNA was isolated and subjected to RT-qPCR using ORF20 gene-specific primers. The ORF59 early gene and ORF65 late gene were used as references. Each reaction was performed in triplicate, and each data point represents the average of three biological repeats. Statistical analysis was performed using ‘HH’ R package, with mean and standard deviation values exported from StepOnePlus software. *p*-values were adjusted using FDR correction for multiple testing. * *p* < 0.05, ** *p* < 0.01, **** *p* < 0.0001.

During the lytic infection cycle, KSHV genes are expressed in a temporally regulated manner, eventually presenting a full repertoire of viral lytic gene products. The lytic genes are categorized into three groups based on their expression kinetics: IE, E, and L [14,15,16]. To determine the kinetics of ORF20 expression during lytic reactivation, we induced lytic reactivation in wild-type (WT) BAC16-infected iSLK cells in the absence or presence of the viral DNA polymerase inhibitor phosphonoacetic acid (PAA), which abrogates expression of late viral genes. The cells were collected at 24, 48, and 72 h post induction and RT-qPCR was used to determine the levels of ORF20 mRNA. The early gene, ORF59, and the late gene, ORF65, were used as reference genes. The expression patterns of ORF20 and ORF65, as depicted in Figure 2, were similar and both displayed sensitivity to PAA. This suggests that ORF20 exhibits late kinetics of expression during lytic induction.

### 3.3. Construction of Recombinant ORF20-Null KSHV Genome

**Figure 3:**
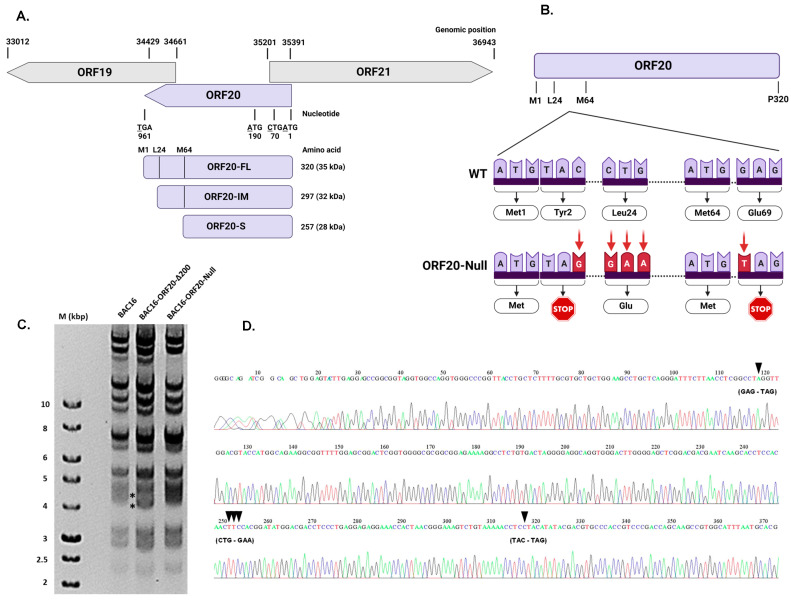
Construction and analysis of BAC16-ORF20-Null. (**A**) Genomic organization of the KSHV ORF19–ORF21 gene cluster. ORF20, which is 960 nucleotides in length, is encoded on the minus-strand of the genome. The neighboring genes, ORF19 and ORF21, are encoded on the minus-strand and the plus-strand, respectively, and partially overlap ORF20. ORF20 encodes three protein isoforms that can be translated from three alternative initiation codons: ORF20-FL, initiating at Methionine 1 (Met 1) (aa 1–320); ORF20-IM, initiating at Leucine 24 (Leu 24) (aa 24–320); and ORF20-S, initiating at Met 64 (aa 64–320). All numbering is according to BAC16 cloned from JSC-1 cells (GenBank Accession Number: GQ994935.1). The calculated molecular weight (MW) for each isoform is indicated on the right. Created with BioRender.com. (**B**) Schematic diagram illustrating wild-type (WT) ORF20 and the resulting mutated ORF20-Null, containing two termination codons and a third point mutation. Created with BioRender.com. (**C**) Restriction fragment length polymorphism analysis of BAC16, the intermediate BAC16-ORF20-Δ200 clone and the resulting BAC16-ORF20-Null. BAC DNAs digested with XhoI and resolved on a 0.4% gold agarose gel stained with ethidium bromide. As predicted, elimination of 200 bp led to a decrease in size of 4456 bp fragment resulting in a 4256 bp band. Subsequent recombination involved the insertion of a synthetic cassette containing kanamycin and the same deleted 200 bp with selected mutations. Following elimination of the kanamycin cassette, a second recombination took place, resulting in a 4456 bp band as predicted. Analysis of BAC16-ORF20-Null tagged with mCherry did not reveal alteration in the restriction pattern. Asterisks indicate altered bands. MW markers (M) are shown on the left. (**D**) Sequence analysis of the recombinant BAC16-ORF20-Null. Black arrowheads indicate the mutated positions, as expected.

Previous studies on HSV-1 and VZV have demonstrated that null mutations of the ORF20 homologs UL24 and ORF35, respectively, result in reduced infectious virus production [59]. To determine the function of KSHV ORF20 during infection, we generated a BAC16-ORF20-Null recombinant carrying the entire KSHV genome, which fails to express the FL, IM, and S isoforms of ORF20. As depicted in Figure 3A, ORF20 is 960 nucleotides in length and is transcribed on the minus strand of the genome. Its neighboring genes, ORF19 and ORF21, are encoded, on the minus- and plus-strands, respectively, and overlap ORF20 on both sides; thus, manipulation of this gene was carefully designed to affect only ORF20. Of note, Hoffman et al. [26] generated a similar mutated KSHV genome by inserting a single stop-codon after the internal Methionine initiation codon. This virus fails to express all ORF20 isoforms, yet in contrast to the mutant described here, it may express a short peptide of 68aa, expected to be included in the N-terminus of ORF20-FL and five residues included in the N-terminus of ORF20-S. To generate the BACmid, we utilized the two-step red-recombination method using BAC16 that constitutively expresses mCherry fluorescent protein [43]. This involved two stages, including generation of intermediate recombinant viruses. First, we deleted a 200 bp fragment, followed by insertion of a synthetic cassette containing the same deleted 200 bp fragment with point mutations that nullify expression of ORF20: termination codons replacing both Tyr2 and Glu69 (TAC to TAG, GAG to TAG) and a mutation replacing Leu24 with Glu (CTG to GAA) (Figure 3B). The resulting BAC was termed “BAC16-ORF20-Null”. We verified the integrity of the viral genome and its terminal repeats by restriction fragment length polymorphism analysis (Figure 3C). In addition, we amplified the ORF20 region by PCR, and sequenced it to confirm the presence of the mutations in the engineered BACmid (Figure 3D).

### 3.4. Lytic Reactivation of ORF20-Null Viruses Results in Accelerated Transcription of Viral Lytic Genes, an Earlier Accumulation of Viral Lytic Proteins, an Increase in the Quantity of Viral DNA Copies and a Decrease in Virion Production

**Figure 4:**
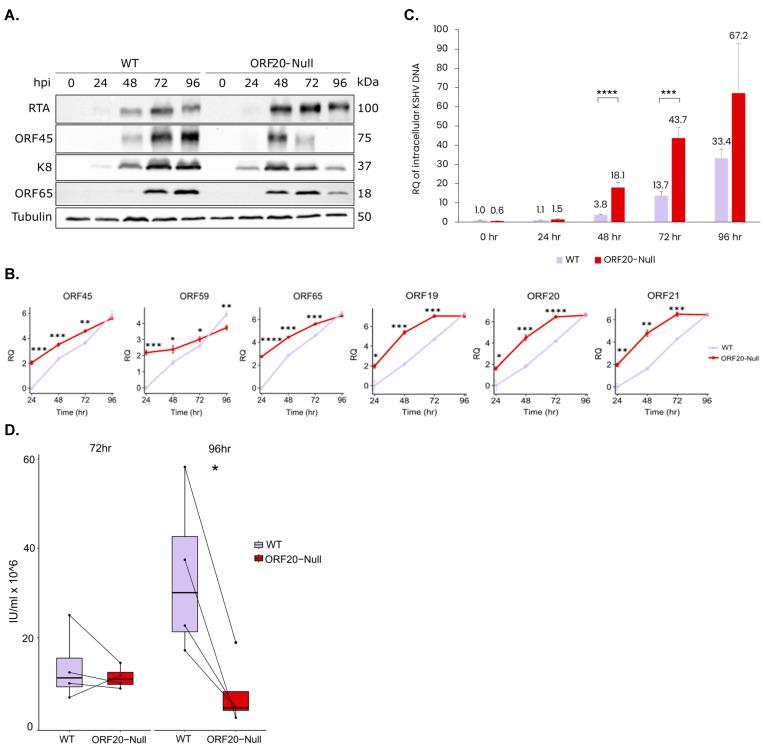
Comparison of protein expression and levels of mRNA, DNA and infectious particle production in wild-type (WT) and ORF20-Null-infected iSLK cells upon lytic induction. iSLK cells harboring WT or ORF20-Null KSHV were induced to undergo lytic virus reactivation using doxycycline and n-Butyrate. (**A**) Whole-cell lysates were prepared at 0, 24, 48, 72, and 96 h post induction (hpi), and protein expression of viral lytic genes was analyzed by immunoblotting using the indicated antibodies. The same blot was also re-probed with anti-Tubulin as a loading control. The immunoblot shown is representative of three biological repeats that yielded similar results. (**B**) RNA was isolated and the levels of ORF45, ORF59, ORF65, ORF19, ORF20, and ORF21 transcripts were measured by RT-qPCR. The relative quantity (RQ) values were log-transformed for statistical analysis and presentation (*Y*-axis). The graphs represent data from three biological repeats. Statistical analysis was performed using ‘HH’ R package, with mean and standard deviation values exported from StepOnePlus software. *p*-values were adjusted using FDR correction for multiple testing. * *p* < 0.05, ** *p* < 0.01, *** *p* < 0.001, **** *p* < 0.0001. (**C**) High-molecular-weight DNA was extracted and quantitative TaqMan PCR assay was performed to amplify the ORFK6 viral gene and the single copy cellular ERV3 gene. The reactions were conducted in triplicate, and the mean values obtained were normalized to the cellular ERV3 gene. Statistical analysis was performed using BSDA R packages, with mean and standard deviation values exported from CFX96 software. *p*-values were adjusted using Bonferroni correction for multiple testing. (**D**) Supernatants were collected 72 and 96 h following lytic induction. Infectious unit (IU) titers were determined based on FACS analysis of mCherry-positive infected cells. Results shown represent the average of four biological repeats. * *p* < 0.05, ** *p* < 0.01, *** *p* < 0.001, **** *p* < 0.0001.

To investigate the role of ORF20 in lytic reactivation of KSHV, we transfected HEK-293T cells with WT BAC16 and BAC16-ORF20-Null DNAs. These cells were then treated to induce lytic reactivation and co-cultivated with iSLK cells that were selected with hygromycin to establish latently infected iSLK cell lines. To compare the lytic reactivation cycle between WT and ORF20-Null viruses, we induced the lytic cycle using doxycycline to induce the expression of RTA, along with n-Butyrate. Proteins were then extracted at 0, 24, 48, 72, and 96 h post induction, and analyzed by Western blot (WB). As shown in Figure 4A, lytic induction of WT-infected cells resulted in accumulation of viral lytic proteins, including the early gene product K8, and the late small capsid protein, ORF65. Accumulation of the ORF45 protein, which can be activated through RTA-dependent and independent pathways [60], was evident as well. Interestingly, lytic induction of cells infected with ORF20-Null viruses led to an earlier accumulation of all tested viral lytic proteins followed by a subsequent decline in their expression. Additionally, we measured the mRNA levels of ORF45, ORF59, and ORF65 transcripts, representing IE, E, and L viral genes, respectively, as well as ORF20 and its overlapping genes, ORF19 and ORF21, using RT-qPCR at 24, 48, 72, and 96 h post induction (Figure 4B). The results showed significant differences in the transcript levels of the lytic genes at early time points, while the gap in their expression levels between WT and ORF20-Null-infected cells closed at later time points. These results suggest that the absence of ORF20 has a broad impact on the kinetics of viral gene expression following lytic reactivation.

To determine whether ORF20 is required for viral DNA replication and for the production of infectious virions, we induced the lytic cycle in iSLK-WT and iSLK-ORF20-Null-infected cells. DNA was extracted from the cells at 0, 24, 48, 72, and 96 h following lytic induction. As shown in Figure 4C, TaqMan PCR revealed a significant increase in the quantity of viral DNA copies in ORF20-Null infected cells, starting 48 h post induction, indicating enhanced viral DNA replication in ORF20-Null-infected cells. Additionally, cell-free viruses were collected from the supernatants 72 and 96 h post induction. Naïve SLK cells were used to quantitate infectious units (IU) based on the percentage of mCherry-positive infected cells using flow cytometry. As shown in Figure 4D, despite the higher yield of viral DNA, cells infected with ORF20-Null viruses produced lower yields of infectious virions compared to WT-infected cells, and this difference was significant at 96 h post induction.

### 3.5. Lack of ORF20 Leads to an Increase in Cell Death at Early Time Points after Lytic Induction

**Figure 5:**
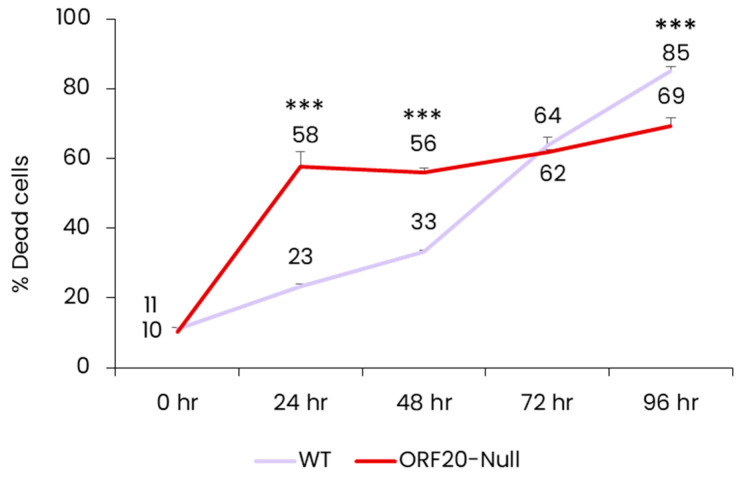
Cell death upon lytic induction in wild-type (WT) and ORF20-Null-infected iSLK cells. Cells were treated with doxycycline and n-Butyrate to induce lytic virus reactivation and collected 0, 24, 48, 72, and 96 h post induction. The extent of cell death was determined by trypan blue uptake. The graph represents three biological repeats. Data were analyzed by two-way ANOVA test followed by Tukey’s HSD post hoc test. *** *p* < 0.001.

During characterization of lytic reactivation of BAC16-ORF20-Null-infected iSLK cells, we noticed that these cells exhibit a higher level of cell death compared to cells infected with WT virus. Cell death plays a crucial role in defense against viral infection as it may hinder virus production, and thereby prevent the spread of the virus from infected cells to uninfected ones. Accordingly, viruses encode various inhibitors that target cell death pathways. To validate our initial observation, we induced the lytic cycle in WT and BAC16-ORF20-Null-infected cells and assessed cell death by trypan blue staining 0, 24, 48, 72, and 96 h post induction. As shown in Figure 5, cells infected with ORF20-Null virus exhibited significantly higher levels of cell death at the early time points post induction compared to cells infected with WT virus. This difference, however, declined over time, while the WT virus showed a steady increase in the percentage of dead cells. This suggests that ORF20 participates in the regulation of cell death during lytic induction while its absence leads to early cell death.

### 3.6. ORF20 with Its Conserved Putative Endonuclease Motif Is Required for Efficient Production of KSHV Infectious Particles

**Figure 6:**
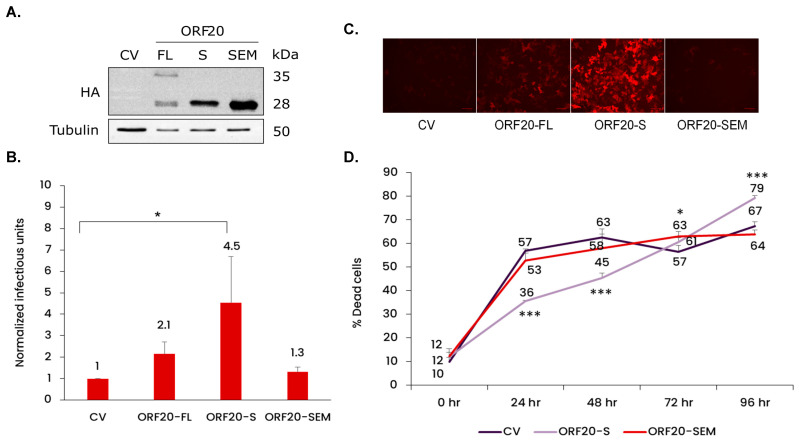
Functional complementation of ORF20-Null KSHV. iSLK-ORF20-Null cells were transduced with 10 MOI of recombinant lentiviruses encoding HA-tagged full length (ORF20-FL), short (ORF20-S), and short endonuclease mutant (ORF20-SEM) or with a control vector (CV) followed by lytic induction 2 h later. (**A**) Cell lysates were prepared after 96 h, separated on SDS-PAGE, and probed with HA antibody. Anti-Tubulin was used for loading control. (**B**) Supernatants were collected 96 h later and used to infect SLK cells. Infectious virus titers were determined based on FACS analysis of mCherry-positive SLK cells. The graph represents four biological repeats. One sample t-test was performed on normalized values, followed by FDR multiple-testing correction: * *p* < 0.05. (**C**) Representative images of mCherry fluorescent reporter expressed in SLK cells captured 48 h following infection with supernatants from ORF20-Null infected iSLK cells transduced with lentiviral vectors according to B. Images were captured using ZOE Fluorescent Cell Imager (Bio-Rad Laboratories, Hercules, Calif.); scale bars, 100 μM. (**D**) The extent of cell death in BAC16-ORF20-Null-infected cells that were treated as described in B was determined by trypan blue uptake. The graph represents three biological repeats. Data were analyzed by two-way ANOVA test followed by Tukey’s HSD post hoc test. * *p* < 0.05, *** *p* < 0.001.

To further investigate the importance of ORF20 in lytic reactivation of KSHV, we constructed recombinant lentiviral vectors encoding full-length ORF20 (ORF20-FL) capable of expressing all three isoforms and the short ORF20 isoform (ORF20-S), both tagged with HA at the C-terminus. In addition, we prepared a lentiviral vector encoding the short isoform with point mutations in the conserved putative UL24 family endonuclease motif (E154A/K156A), referred to as ORF20-Short-Endonuclease-Mutant (ORF20-SEM). The resulting lentiviral vectors were transduced into iSLK-ORF20-Null cells followed by lytic reactivation. To validate protein expression of the various HA-tagged ORF20 isoforms and to ensure lentiviral expression during lytic reactivation, cell lysates were collected 96 h post induction and examined by WB analysis. The anticipated molecular weights (MWs) of the proteins generated from the FL form were 35, 32, and 28 kDa, while the S and SEM vectors were expected to express 28 kDa HA-tagged proteins. As shown in Figure 6A, although the vector encoding ORF20-FL was expected to express the FL, IM, and S isoforms, only two bands of 35 and 28 kDa, corresponding to the long and short isoforms, were detected. This could be due either to the level of protein expression, or to the context of the gene within our vector as compared with its position within the viral genome. The ORF20-S and ORF20-SEM constructs, expected to express only the S isoform, each produced distinct bands with MWs similar to the lower MW band observed with ORF20-FL vector. Of note, the expression levels of the different isoforms varied, with ORF20-S and ORF20-SEM repeatedly demonstrating expression at particularly high levels.

Functional complementation utilized transduction of the different ORF20 vectors into iSLK-ORF20-Null cells followed by lytic induction. The resulting viral particles were collected from the supernatant 96 h post induction and IU were quantified using SLK cells based on the expression of mCherry. As shown in Figure 6B,C, expression of the ORF20-S isoform significantly increased infectious virus production upon lytic induction. In contrast, the mutated short ORF20 isoform, ORF20-SEM, failed to complement ORF20 deficiency, indicating that this motif is necessary for the function of ORF20 during lytic reactivation. We also evaluated cell death through trypan blue staining at 0, 24, 48, 72, and 96 h post transduction and induction. As illustrated in Figure 6D, iSLK-ORF20-Null cells transduced with CV or ORF20-SEM exhibited significantly elevated levels of cell death during the initial time points compared to cells transduced with ORF20-S. These findings collectively suggest that ORF20 protein supports efficient lytic reactivation, and that its putative endonuclease motif is essential for its activity.

### 3.7. ORF20 Is Required for IL6 and CXCL8 Induction by KSHV

**Figure 7:**
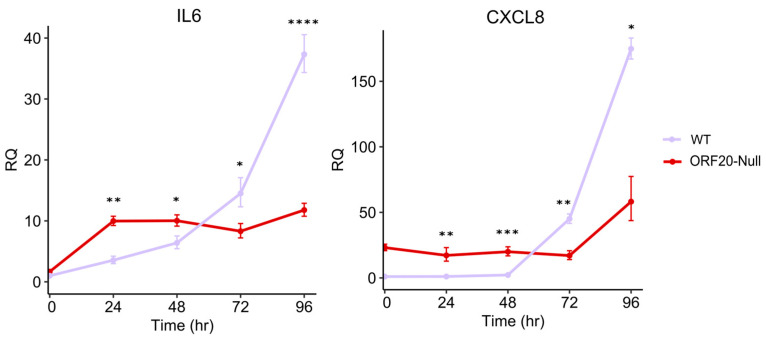
IL6 and CXCL8 transcript levels following lytic induction of wild-type (WT) and ORF20-Null viruses. iSLK-WT and iSLK-ORF20-Null cells were induced to undergo lytic reactivation and collected at 0, 24, 48, 72, and 96 h post induction. RNA was extracted and subjected to real-time RT-PCR. IL6 and CXCL8 transcript levels were examined in triplicate, and the results obtained were normalized to GAPDH and presented relative to WT 0 h. Statistical analysis was performed using ‘HH’ R package, with mean and standard deviation values exported from StepOnePlus software. * *p* < 0.05, ** *p* < 0.01, *** *p* < 0.001, **** *p* < 0.0001.

Previous studies have shown that expression of UL76, the hCMV ORF20 homolog, activates DNA damage response signaling, resulting in increased γ-H2AX foci formation in nuclei [23]. This response leads to the activation of the NF-kB pathway, and induction of C-X-C motif chemokine ligand 8 (CXCL8) expression mediated by the ATM kinase [61]. In contrast, Tong-Yun Wang et al. reported that PRV UL24 dampens TNF-α-mediated NF-κB activation and significantly reduces interleukin 6 (IL6) and CXCL8 expression [38]. Given these contradicting functions, we aimed to determine the potential involvement of ORF20 in the NF-kB pathway. Therefore, we evaluated the impact of ORF20 on the expression of IL6 and CXCL8. iSLK-WT and iSLK-ORF20-Null cells were induced to undergo lytic reactivation, and cells were collected at 0, 24, 48, 72, and 96 h post induction. RT-qPCR analysis was used to determine the levels of IL6 and CXCL8 transcripts. Transient increased expression of IL6 and CXCL8 was observed following lytic induction (Figure 7); however, the mRNA levels of both genes were significantly higher at early time points and lower at later time points post induction in BAC16-ORF20-Null-infected cells compared to the WT, suggesting the involvement of ORF20 in the regulation of the expression of these genes following lytic induction, possibly through activation of the NF-kB pathway.

## 4. Discussion

The KSHV encoded ORF20 belongs to the UL24 gene family and is conserved in all three herpesvirus subfamilies. The UL24 gene family shares five domains of sequence similarity, and a putative endonuclease motif, PD-(D/E)XK, whose enzymatic activity has not yet been verified [19]. According to next-generation ribosomal footprinting data, ribosomes bind at two positions, giving rise to two co-linear ORF20 isoforms, the FL isoform and the IM isoform. A third S isoform starting at an internal Methionine is predicted in silico [20]. As shown in Figure 1, the N-terminal amino acids included in ORF20-FL are unique to KSHV, suggesting that the short ORF20-S isoform is likely to manifest the conserved functions of this protein family, while the two other isoforms may have other functions.

In the present study, we aimed to increase our understanding of the function of the poorly characterized KSHV ORF20 protein. MHV68 ORF20 is characterized by early-late kinetics [62], similar to PRV UL24 which is also expressed during later stages [63]. We therefore used PAA to generate stage-specific RNA to identify the kinetic class of KSHV ORF20 in iSLK cells. As reference, we employed ORF59 and ORF65 genes with known kinetics representing early and late viral genes, respectively (Figure 2). Our analysis revealed that ORF20 gene has late expression kinetics in SLK cells. This supports the classification of ORF20 as a late gene, as shown in a study on the kinetic profiling of the expression of KSHV proteins in endothelial cells, in which ORF65 and ORF20 were classified in the same cluster whose expression increases in later times following lytic reactivation (48–72 h) and were markedly PAA sensitive [64].

Our study also aimed to determine the importance of ORF20 during KSHV infection. Based on the complete BAC16 KSHV genome, we generated a recombinant virus that fails to express all three ORF20 isoforms. Using this construct, we demonstrate that KSHV ORF20 participates in coordinating efficient lytic virus reactivation for enhanced infectious viral yield. Lytic reactivation of ORF20-Null viruses resulted in an earlier accumulation of viral lytic proteins compared to WT virus, evident 24 h post reactivation (Figure 4A). In line with this, RT-qPCR analysis showed accelerated accumulation of ORF45, ORF59, and ORF65 lytic viral transcripts at early time points, as well as ORF20 and its neighboring genes ORF19 and ORF21, with the gap closing at later time points, indicating that the absence of ORF20 has a broad impact on viral gene expression following lytic reactivation (Figure 4B). Furthermore, lack of ORF20 expression decreased viral yield of KSHV (Figure 4D), although viral DNA levels were higher in ORF20-Null infected cells, possibly due to the delayed viral assembly (Figure 4C). Additionally, ORF20-Null virus-infected cells exhibited significantly higher cell death at early time points, compared to WT virus-infected cells (Figure 5). This finding reinforces the link between UL24 homologs and the regulation of cell death, as previously demonstrated upon their ectopic expression [21,22]. Of note, Hoffman et al. constructed a KSHV ORF20-stop mutant, which unlike the mutant described here, allows expression of the first sixty-eight amino acids of the long isoform and five amino acids of the short isoform. Similar to our findings, they also observed a significant impairment in the production of infectious virions compared to WT infected cells [26]. However, their analysis was conducted at a single time point, precluding the identification of differences in kinetics upon lytic reactivation, including accelerated viral gene transcription and the early accumulation of lytic proteins. Additionally, we revealed early cell death during lytic reactivation of ORF20-Null infected cells, indicating the potential involvement of ORF20 in the regulation of cell death.

Functional complementation of the ORF20-Null mutant virus with ORF20-FL resulted in an insignificant, yet two-fold, increase in virus titers relative to the control vector, while expression of the ORF20-S isoform rescued KSHV production upon lytic induction. In contrast, the endonuclease mutant form of ORF20-S (ORF20-SEM) failed to complement lytic reactivation (Figure 6B,C). Furthermore, functional complementation with ORF20-S reduced early cell death during lytic induction (Figure 6D). Thus, our findings suggest that ORF20, containing the putative endonuclease motif, is important for efficient virus production. The significance of the conserved endonuclease motif of KSHV ORF20 is in line with previous findings regarding its homolog encoded by HSV-1 [56,65,66]. Likewise, hCMV which lacks the entire UL76 gene exhibits a complete loss of virus production [67], while transposon mutagenesis within UL76 resulted in a significant reduction in virus production [68]. Finally, the VZV homolog of UL24, ORF35, is not essential for VZV replication in vitro; however, viral mutants lacking ORF35 produced smaller plaques and exhibited slower growth, resulting in reduced virus yields [69].

Finally, in a study conducted by Costa et al. it was demonstrated that infection with an hCMV mutant virus lacking the UL76 gene leads to an increase in CXCL8 expression levels [61]. This finding holds particular significance since CXCL8 promotes hCMV replication and facilitates efficient viral spread by neutrophils [70,71]. It is believed that the transmission of hCMV between infected epithelial cells or fibroblasts in the tissues to endothelial cells is responsible for virus dissemination through the bloodstream [72]. We therefore examined whether ORF20 elicits a similar impact as UL76, which prompts a DNA damage response mediated by ATM kinase and triggers the NF-κB pathway, leading to the expression of CXCL8. We found that lytic reactivation of ORF20-null virus results in increased expression of CXCL8 and IL6, whereas the levels of these transcripts are lower at later time points during which virus release and dissemination take place. This effect may be due to ORF20 directly affecting the transcription of CXCL8 and IL6 genes, or indirectly due to changes in the lytic replication kinetics (Figure 7).

Taken together, our findings provide further evidence of the role of ORF20 in coordinating efficient viral gene expression, DNA replication, and infectious viral production. This may result from a potential involvement of ORF20 in modulating cell death dynamics during viral lytic activation, which in turn controls virus propagation. Our results indicate that the short ORF20 isoform, containing the conserved putative endonuclease motif, is sufficient for efficient lytic reactivation. However, the unique functions of the other two ORF20 isoforms, and whether the UL24 protein family possesses an inherent endonuclease activity, remain to be determined.

## Data Availability

There is no additional data to add.
